# Bioactive Properties of Hazelnut-Derived Products in Colorectal Cancer Prevention: A Systematic Review of Preclinical and Epidemiological Studies

**DOI:** 10.3390/foods14132154

**Published:** 2025-06-20

**Authors:** Giuseppe Mazzola, Mariangela Rondanelli, Federico Buga, Patrizia Riso, Simone Perna

**Affiliations:** 1 Endocrinology and Nutrition Unit, Azienda di Servizi alla Persona “Istituto Santa Margherita”, University of Pavia, 27100 Pavia, Italy; 2 Department of Public Health, Experimental and Forensic Medicine, University of Pavia, 27100 Pavia, Italy; mariangela.rondanelli@unipv.it; 3 Department of Food, Environmental and Nutritional Sciences, Division of Human Nutrition, University of Milan, 20133 Milan, Italy; federico.buga@studenti.unimi.it (F.B.); patrizia.riso@unimi.it (P.R.); simone.perna@unimi.it (S.P.)

**Keywords:** hazelnut, colorectal cancer, apoptosis, bile acids, oxidative stress, fermentation, fiber, polyphenols, nutritional oncology

## Abstract

**Background**: Colorectal cancer (CRC) is among the leading causes of cancer-related mortality worldwide, with increasing attention being paid to modifiable dietary factors in its prevention. Hazelnut (*Corylus avellana* L.) represent a nutrient-dense food rich in unsaturated fats, polyphenols, fiber, and phytosterols, with potential anticarcinogenic properties. This systematic review aimed to evaluate the role of hazelnut consumption in the prevention and modulation of CRC risk, with specific focus on experimental, mechanistic, and preclinical evidence. **Methods**: Following PRISMA guidelines, a systematic search was conducted in PubMed, Google Scholar, and the Cochrane Library for articles published from 2015 onward. Eligible studies included original in vitro and in vivo models, as well as observational studies, evaluating hazelnut or hazelnut-derived products in relation to CRC-related biological, metabolic, or clinical outcomes. Data extraction focused on bioactive composition, experimental models, molecular pathways, and fecal/metabolic markers of carcinogenesis. **Results**: A total of 11 studies were included after screening 24 records: 8 in vitro investigations, 2 in vivo animal experiments, and 1 epidemiological study. In vitro studies showed that hazelnut derivatives—including fermented hazelnuts and oil-based extracts—exert antiproliferative effects via BAX/BCL-2 modulation, increased caspase-3 activity, and oxidative stress reduction. In vivo studies confirmed improved lipid metabolism, modulation of bile acid composition (notably reduced lithocholic/deoxycholic acid ratio), and enhanced antioxidant defenses. FIBEROX^®^, a hazelnut skin extract enriched in dietary fiber, demonstrated promising effects on gut microbiota and bile acid detoxification. **Conclusions:** Hazelnut and their bioactive compounds may aid CRC prevention through multiple molecular and metabolic pathways. Further human studies are needed to confirm these effects and support dietary recommendations.

## 1. Introduction

Colorectal cancer (CRC) is one of the most common and deadly malignancies globally, causing nearly 900,000 deaths per year [1]. Its rising incidence is largely attributed to aging populations and modifiable risk factors such as unhealthy dietary patterns, physical inactivity, obesity, and smoking [2]. In Italy, CRC is the second most diagnosed cancer, with approximately 43,700 new cases and over 19,000 deaths annually. National screening programs and multidisciplinary therapeutic approaches have improved early detection and patient outcomes, leading to a five-year survival rate of around 65% [3]. Nonetheless, symptoms often emerge at advanced stages, reinforcing the need for preventive strategies centered on dietary interventions.

Within this framework, hazelnuts (*Corylus avellana* L.) have emerged as a promising candidate due to their high content of bioactive compounds with potential anticancer properties. These include oleic acid, polyunsaturated fatty acids, tocopherols, phytosterols, polyphenols, and L-arginine, all of which are known to exert antioxidant, anti-inflammatory, and cholesterol-lowering effects [4,5]. Moreover, hazelnut skins—a major byproduct of roasting—contain even higher levels of polyphenols than the kernels themselves and are being explored for their nutraceutical potential [6,7].

Recent advances in the management of CRC have significantly improved patient outcomes, particularly through the integration of multimodal treatment strategies that include endoscopic resection, surgery, radiotherapy, chemotherapy, immunotherapy, and targeted biological agents [1,2]. For non-metastatic cases, surgical excision combined with neoadjuvant or adjuvant therapy remains the standard approach, while advanced CRC may be treated with systemic regimens such as FOLFOX or FOLFIRI protocols, often combined with anti-EGFR (e.g., cetuximab) or anti-VEGF (e.g., bevacizumab) monoclonal antibodies [8]. Moreover, immune checkpoint inhibitors targeting PD-1 or CTLA-4 have shown efficacy in selected molecular subtypes, particularly microsatellite instability-high (MSI-H) tumors [1]. Despite these advances, the long-term management of CRC still faces challenges related to therapy resistance, toxicity, and residual inflammation. In this context, interest has grown around the use of dietary components and functional foods as adjunctive strategies to modulate key carcinogenic pathways [7]. Hazelnut and their derivatives have demonstrated the ability to influence molecular targets relevant to CRC pathogenesis, including apoptosis regulation, oxidative stress mitigation, bile acid metabolism, and inflammatory signaling [6,9,10].

In vitro and in vivo studies—discussed in detail in the Results section—suggest that hazelnut oil, skins, and fermented extracts contain bioactive compounds capable of interacting with key cellular pathways also targeted by pharmacological agents. These include modulation of mitochondrial apoptotic regulators (e.g., BAX/BCL-2), caspase activation, enhancement of antioxidant enzymes (e.g., SOD2, GSTP1), and rebalancing of gut–liver axis biomarkers such as bile acids [11,12,13]. Such properties support the hypothesis that hazelnuts may exert complementary effects to standard therapies, especially in the prevention or reduction of early-stage carcinogenic processes. However, current epidemiological data on hazelnut consumption and CRC risk remain limited and inconclusive, and are mostly derived from studies investigating total “tree nut” intake without distinguishing among nut types or preparations. The specific role of hazelnuts in modulating cancer risk in humans remains to be elucidated [14].

The aim of this systematic review is to critically examine the potential protective effects of hazelnut consumption against colorectal cancer, focusing on mechanistic, preclinical, and observational evidence. In addition, we explore the relevance of hazelnut byproducts, such as skins and oil, as sustainable and functional food ingredients. This review provides a rationale for future translational research by critically appraising preclinical evidence on hazelnut-derived products and identifying their mechanistic role in colorectal cancer prevention.

## 2. Materials and Methods

### 2.1. Design and Objectives

This systematic review was conducted in accordance with the PRISMA 2020 guidelines [15], with a specific focus on preclinical evidence. The aim was to evaluate the effects of hazelnut-derived compounds—primarily tested in vitro and in vivo—on molecular and physiological markers of colorectal carcinogenesis. The primary objective was to synthesize experimental and mechanistic evidence from in vitro and in vivo models, and to assess whether preliminary human data provide evidence of a protective role of hazelnut-derived compounds in CRC.

### 2.2. Literature Search Strategy

A comprehensive literature search was performed using three electronic databases, PubMed, Google Scholar, and the Cochrane Library, covering the period from January 2015 to December 2024. The search included only English-language articles published in peer-reviewed journals.

The search strategy combined Medical Subject Headings (MeSH) and free-text terms related to hazelnuts, colorectal cancer, and relevant molecular or metabolic mechanisms, using the following Boolean structure:

*“Corylus avellana” OR “hazelnuts” OR “hazelnut oil” OR “hazelnut skin” AND “colorectal cancer” OR “colorectal neoplasms” OR “colon cancer” OR “rectal cancer” AND “apoptosis” OR “oxidative stress” OR “bile acids” OR “inflammation” AND “nutritional properties” OR “dietary fiber” OR “flavan-3-ols” OR “FIBEROX^®^”*

Additional studies were identified by screening reference lists of the included articles.

### 2.3. Inclusion and Exclusion Criteria

Studies were deemed eligible if they met the following criteria:

- Involved in vitro, in vivo, or observational human models relevant to CRC;
- Assessed the effects of hazelnuts or hazelnut-derived compounds (including oils, skins, or fermented extracts);
- Reported at least one outcome related to CRC risk, tumorigenesis, or biological mechanisms involved in carcinogenesis (e.g., bile acid metabolism, oxidative stress, inflammatory pathways, apoptosis);
- Were original research articles published in full text in English.

Exclusion criteria included the following:

- Narrative reviews, conference proceedings, editorials, or letters;
- Studies focusing on general “tree nut” consumption without hazelnut-specific data;
- Articles lacking CRC-relevant outcomes or mechanistic endpoints.

### 2.4. Study Selection and Data Synthesis

All identified records were imported into a reference management software, and duplicates were removed. Two independent reviewers screened the titles and abstracts. Full texts of potentially relevant studies were retrieved and assessed for eligibility. Disagreements were resolved through consensus. Descriptive analyses and tabular summaries of extracted variables were performed using IBM SPSS Statistics (version 28.0; IBM Corp., Armonk, NY, USA), which facilitated structured data handling and interpretation. No meta-analytical procedures were applied due to the methodological heterogeneity of the included studies.

Given the heterogeneity of study designs, intervention types, and outcome measures, no meta-analysis was conducted. A qualitative synthesis was performed instead. Studies were grouped into three categories based on methodological approach: in vitro studies, in vivo studies, and epidemiological studies.

**Figure 1:**
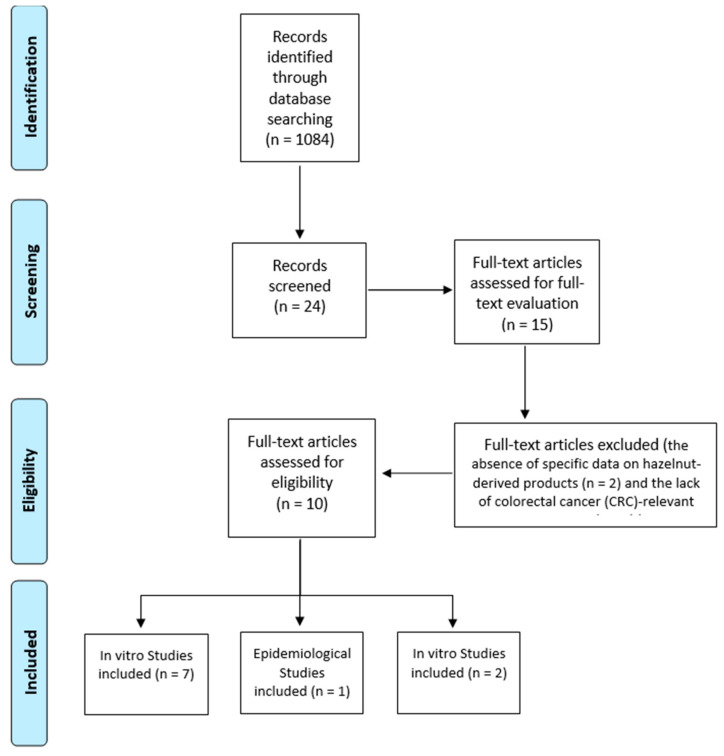
PRISMA flow diagram of selected studies.

The results are reported narratively and supported by structured summary tables. A PRISMA flow diagram detailing the study selection process is shown in Figure 1.

## 3. Results

The initial database search yielded 1084 records. After removal of duplicates (*n* = 853), 231 articles were screened based on title and abstract. Among these, 207 were excluded for not meeting the inclusion criteria (e.g., lacking hazelnut-specific data, not related to CRC, or not original research), leaving 24 articles for full-text review. After duplicate removal and preliminary screening of titles and abstracts, 15 articles were retrieved for full-text evaluation. Following the application of predefined inclusion and exclusion criteria, 10 studies were ultimately included in the qualitative synthesis. The main reasons for exclusion were the absence of specific data on hazelnut-derived products (*n* = 2) and the lack of colorectal cancer (CRC)-relevant outcomes (*n* = 2). Of the included studies, 7 were in vitro investigations conducted on CRC cell lines, 2 were in vivo experiments in animal models of colorectal carcinogenesis or metabolic disease, and 1 was an epidemiological study analyzing nut consumption and CRC incidence in human populations. The PRISMA flow diagram summarizing the study selection process is presented in Figure 1.

### 3.1. In Vitro Studies

The in vitro studies selected for this review are summarized in Table 1 and the related risk of bias is presented in Table 2. Overall, the studies differed based on the type of hazelnut derivative tested (titrated extracts versus non-titrated extracts) and the origin of the cell lines used (human-derived versus animal-derived).

#### 3.1.1. Cancer Biology Context: Hazelnut Extracts and Colorectal Cancer (CRC)

As shown in Table 1, based on in vitro studies investigating the effects of hazelnut-derived products on colorectal cancer models, hazelnut’s antioxidant-rich compounds have gained attention for their potential to modulate these pathways and exert chemopreventive or therapeutic effects.

#### 3.1.2. Fermented Hazelnut Skins and Antioxidant Defense Mechanisms

The study by Schlörmann and colleagues [11] investigated how fermentation alters the bioactivity of hazelnut skins—byproducts rich in polyphenols and other phytochemicals—and their impact on oxidative stress and DNA damage in HT29 human colon carcinoma cells. The results showed that fermentation significantly enhanced the biological activity of these extracts, likely due to increased bioavailability and transformation of phenolic compounds into more active metabolites. In particular, fermented hazelnut skins induced the expression of key antioxidant enzymes, including the following: SOD2 (superoxide dismutase 2): This enzyme catalyzes the conversion of superoxide radicals into hydrogen peroxide and oxygen, playing a critical role in mitochondrial redox homeostasis. GSTP1 (glutathione S-transferase pi 1): Part of the glutathione system, GSTP1 detoxifies reactive electrophilic compounds and protects cells from oxidative damage. This upregulation leads to a significant decrease in intracellular ROS levels and oxidative DNA damage, independent of roasting treatment. These findings suggest that fermentation enhances the ability of hazelnut skin extracts to activate the Nrf2 (nuclear factor erythroid 2-related factor 2) pathway, a master regulator of the cellular antioxidant response. Activation of Nrf2 leads to transcriptional activation of genes encoding detoxifying and antioxidant enzymes, thus protecting cells from oxidative injury and potentially preventing malignant transformation or slowing tumor growth.

#### 3.1.3. Hazelnut Oil and Mitochondrial Apoptotic Pathways

In contrast, Ramezan and colleagues [10] focused on the effects of hazelnut oil on mitochondrial-mediated apoptosis in HCT116 colorectal cancer cells. Apoptosis, or programmed cell death, is another crucial mechanism dysregulated in cancer. Tumor cells often evade apoptosis through overexpression of anti-apoptotic proteins or downregulation of pro-apoptotic signals.

#### 3.1.4. Comparative Effects of Non-Titrated vs. Titrated Extracts

Studies using non-titrated or crude hazelnut derivatives, such as those by Gleiand colleagues [9], demonstrated less pronounced effects compared to fermented products. While non-fermented hazelnut skins also increased detoxifying enzyme activity, they were less effective in reducing ROS levels and oxidative DNA damage. This highlights the importance of standardized extraction and processing techniques, such as fermentation, in enhancing the bioavailability and efficacy of bioactive compounds. The lack of standardization in non-titrated extracts may result in variable concentrations of active constituents, limiting reproducibility and biological impact. Thus, treatment titration and controlled fermentation are critical for optimizing the anticancer potential of natural products like hazelnut extracts.

#### 3.1.5. Use of Human-Derived CRC Cell Lines

All studies referenced utilized human-derived CRC cell lines (e.g., HT29 and HCT116), which are well-established models in cancer research due to their relevance to human pathophysiology. HT29 cells are mucin-secreting, moderately differentiated adenocarcinoma cells, while HCT116 cells are poorly differentiated and exhibit microsatellite instability, making them useful for studying different aspects of CRC biology. However, the exclusive use of human cell lines limits the ability to perform interspecies comparisons or evaluate systemic effects in vivo. Murine models could provide additional insights into the pharmacokinetics, immune modulation, and overall safety profile of hazelnut-derived treatments.

In summary, in vitro evidence suggests that titrated and fermented hazelnut-derived extracts exhibit significantly stronger anticancer properties in colorectal cancer (CRC) cell lines compared to non-titrated products. The enhanced bioactivity of fermented extracts has been attributed to the biotransformation of phytochemicals during fermentation, which increases the concentration and bioavailability of key bioactive compounds such as polyphenols and flavonoids. The main mechanisms involved include the induction of mitochondrial apoptosis, as evidenced by increased caspase-3 activation and mitochondrial membrane potential disruption, enhancement of endogenous antioxidant defenses through upregulation of enzymes like superoxide dismutase (SOD) and catalase, and a marked reduction in oxidative DNA damage, as shown by decreased levels of 8-oxo-dG in treated CRC cells. Importantly, these effects appear to be independent of the roasting process and are specific to human colorectal cancer cells, suggesting a targeted anticancer action.

### 3.2. In Vivo and Epidemiological Studies

The two in vivo studies are summarized in Table 3 (related risk of bias is summarized in Table 4) and the epidemiological study is summarized in Table 5 (related risk of bias is summarized in Table 6).

The only study identified, conducted by Nieuwenhuis and colleagues [14], analyzed the association between tree nut consumption and CRC incidence in the large-scale prospective Netherlands Cohort Study (NLCS). In this cohort, hazelnut-specific intake was not isolated, and total tree nut consumption showed no significant association with overall CRC risk. A weak inverse relationship was observed only in microsatellite instability (MSI) tumors among women, suggesting a possible subtype-specific protective effect [14].

Regarding the in vivo studies, both studies focused on the impact of hazelnut skin extracts, particularly FIBEROX^®^, a polyphenol- and fiber-enriched formulation derived from roasted hazelnut skins. Caimari and colleagues [12] investigated the effects of FIBEROX^®^ supplementation in hyperlipidemic Golden Syrian hamsters. Administration of FIBEROX^®^ led to significant improvements in plasma lipid profiles, reductions in total cholesterol and triglyceride levels, and a marked decrease in glycemia. Importantly, the intervention modulated fecal bile acid composition, specifically reducing the lithocholic/deoxycholic acid ratio, which is considered a biomarker of increased colorectal carcinogenesis risk [12]. Complementary findings were reported by another preclinical study using a similar hazelnut skin extract, although variations in the experimental design limited direct comparability. In both cases, the modulation of bile acid metabolism was proposed as a key mechanism linking hazelnut bioactives to reduced CRC risk, alongside metabolic improvements that could indirectly influence carcinogenic processes through inflammation and insulin resistance. In contrast to the experimental animal data, the epidemiological evidence remains limited and less conclusive.

A comparison between preclinical and epidemiological findings highlights notable divergences. While in vivo models consistently demonstrate beneficial metabolic and carcinogenic modulation following hazelnut-derived interventions, human data do not currently confirm a clear preventive association. Several factors may account for this discrepancy, including differences in dosage and exposure duration, variations in dietary patterns, lack of specificity for hazelnut intake in epidemiological analyses, and confounding lifestyle factors in human populations. Moreover, while animal studies allowed precise administration of standardized extracts such as FIBEROX^®^, epidemiological studies predominantly assessed general nut consumption without detailed characterization of hazelnut-specific bioactive intake. This lack of granularity may obscure potential benefits attributable to hazelnuts alone.

In summary, the in vivo evidence suggests that hazelnut bioactives, particularly polyphenols and fiber, can positively modulate lipid metabolism, bile acid profiles, and glycemic control, thereby potentially reducing CRC risk factors. However, epidemiological data remain insufficient to establish a direct causal relationship, emphasizing the need for further targeted human intervention studies to clarify the protective role of hazelnuts against colorectal carcinogenesis.

## 4. Discussion

The in vitro studies collectively highlighted the ability of hazelnut derivatives—including oils, whole skins, and fermented products—to modulate critical carcinogenic pathways in colorectal cancer (CRC) cell lines. These effects were mainly characterized by the upregulation of pro-apoptotic markers (e.g., BAX), downregulation of anti-apoptotic proteins (e.g., BCL-2), activation of caspase-3, enhancement of antioxidant enzymes such as SOD2, and stimulation of detoxification systems like GSTP1 [9,10,11,13]. Notably, these findings were consistent across both human- and animal-derived CRC cell lines and appeared independent of the specific formulation used, although titrated extracts exhibited greater reproducibility of results compared to non-titrated ones. Conversely, the in vivo evidence was more limited, predominantly based on a small number of animal studies and epidemiological data that mainly referred to overall “tree nut” consumption rather than hazelnuts specifically [14]. Although supplementation with hazelnut skin extracts such as FIBEROX^®^ demonstrated beneficial effects on bile acid profiles and metabolic markers in animal models [12], epidemiological studies in humans failed to establish a clear protective association between hazelnut intake and CRC risk. Several physiological and biomolecular mechanisms could explain these discrepancies. First, the bioavailability of polyphenols and other functional compounds is markedly reduced in vivo due to gastrointestinal digestion, hepatic first-pass metabolism, and systemic dilution [20]. Second, the gut microbiota—critical for the biotransformation of polyphenols into bioactive metabolites—differs substantially between animal models and human subjects, introducing variability that cannot be captured in vitro [21]. Third, in vitro models often expose cells to concentrations of bioactive compounds that are unattainable through normal dietary intake, potentially overestimating biological effects [20].

Moreover, the systemic inflammatory milieu and tumor microenvironment present in vivo introduce complex interactions between cancer cells, immune cells, and stromal elements that are absent in vitro, influencing the final carcinogenic outcome [20]. The antioxidant and anti-inflammatory effects observed in vitro might thus be mitigated or amplified in living organisms depending on individual metabolic and immune responses. Furthermore, emerging evidence suggests that hazelnut-derived polyphenols may also modulate key oncogenic pathways such as Wnt/β-catenin and NF-κB signaling, both critically involved in colorectal tumorigenesis [20,21]. This potential interaction reinforces the biological plausibility of a protective effect against CRC progression. Additionally, it must be considered that the fermentation processes enhancing hazelnut bioactivity in vitro are unlikely to be replicated naturally within the human gastrointestinal tract, leading to potential differences in the potency of observed effects. Furthermore, interindividual variability in polyphenol absorption, enzymatic polymorphisms, and the composition of the gut microbiota may further modulate the systemic bioavailability and efficacy of hazelnut-derived compounds. These factors highlight the necessity for translational studies that bridge in vitro findings with clinically relevant human outcomes.

### 4.1. Study Limitations

Several limitations must be acknowledged when interpreting the present findings. First, the scarcity of human interventional studies specifically addressing hazelnut consumption and CRC risk limits the generalizability of the results. Second, most epidemiological data amalgamate hazelnuts with other “tree nuts”, precluding the isolation of hazelnut-specific effects. Third, substantial heterogeneity exists across in vitro and in vivo experimental designs, including variations in the type, dose, and formulation of hazelnut derivatives used, as well as in the cellular or animal models employed. Additionally, potential publication bias cannot be excluded, as studies reporting significant beneficial effects may be preferentially published. The limited sample size of available studies also raises concerns regarding the robustness of the conclusions drawn. Differences in experimental protocols, such as the use of different extraction methods or the lack of standardized hazelnut formulations, further complicate the comparison across studies. Moreover, the short duration of most in vivo experiments limits the ability to extrapolate findings to chronic exposure scenarios relevant to human CRC development. Moreover, most of the in vitro and in vivo studies employed enriched hazelnut extracts with concentrations of bioactive compounds that are unlikely to be achieved through regular dietary intake, thereby limiting the direct applicability of these findings to real-world nutritional settings. Finally, epidemiological studies included often relied on self-reported dietary intake data, which is prone to recall bias and measurement inaccuracies, thereby potentially masking true associations.

### 4.2. Clinical Implications and Future Research Directions

The preliminary evidence from experimental studies suggests that hazelnut derivatives may represent a valuable adjunct in the modulation of key pathways involved in CRC carcinogenesis, such as mitochondrial apoptosis regulation, oxidative stress mitigation, and bile acid metabolism rebalancing. Given the increasing focus on personalized and integrative oncology approaches, dietary strategies incorporating hazelnuts could complement conventional therapies, particularly in early-stage disease or prevention settings.

Future research should prioritize well-designed randomized controlled trials evaluating standardized hazelnut interventions with defined dosages and durations, employing intermediate biomarkers as well as clinical endpoints. Such trials should aim to characterize not only clinical efficacy but also safety profiles, especially in populations at high risk for colorectal cancer. Furthermore, mechanistic studies exploring the role of the gut microbiota in the biotransformation and bioefficacy of hazelnut bioactives are warranted, as are investigations into potential synergistic effects with pharmacological agents, particularly immune checkpoint inhibitors or bile acid modulators. The development of nutraceutical formulations based on fermented or bio-enhanced hazelnut components may provide an innovative strategy for maximizing bioactive absorption and therapeutic efficacy. Additionally, the employment of advanced in vitro models such as microphysiological systems and organ-on-chip platforms—especially cancer tumor-on-chip models—should be considered to more accurately assess the toxicity and therapeutic potential of hazelnuts and their byproducts. These cutting-edge technologies offer physiologically relevant insights that bridge the gap between conventional cell culture and in vivo studies, thereby enhancing translational research in this field. Finally, integrating omics technologies, such as metabolomics and microbiomics, into future trials could offer a more comprehensive understanding of individual responses to hazelnut-based interventions. [9,10,11,13].

## 5. Conclusions

The current systematic review highlights the emerging potential of hazelnut-derived products as modulators of key molecular pathways implicated in colorectal cancer (CRC) development. In vitro studies consistently demonstrate that hazelnut oils, skins, and fermented extracts promote apoptosis, reduce oxidative stress, and enhance cellular detoxification mechanisms in colorectal cancer models. In vivo studies further support these findings, although data remain limited and predominantly confined to animal models. Despite promising mechanistic evidence, the lack of robust human epidemiological data specifically addressing hazelnut consumption and CRC risk prevents definitive conclusions. Differences in bioavailability, metabolism, and experimental designs may partially explain the discrepancies between preclinical findings and observational studies.

Nevertheless, the bioactive profile of hazelnuts, particularly their rich content of polyphenols and dietary fiber, supports their consideration within broader dietary strategies aimed at colorectal cancer prevention. Future research should prioritize well-designed clinical trials and mechanistic studies to bridge the translational gap and better characterize the preventive potential of hazelnut-based interventions. Importantly, integrating advanced omics technologies, such as metabolomics and microbiomics, into future trials could offer a more comprehensive understanding of individual responses to hazelnut-based interventions. A more detailed understanding of the interactions between hazelnut bioactives, gut microbiota, and host metabolic pathways will be essential to identify their potential clinical application in oncology nutrition.

Considering their favorable nutritional profile and the mechanistic evidence available, hazelnuts could be incorporated into evidence-based dietary advice to promote colorectal health.

## Figures and Tables

**Table 1:** In vitro studies investigating the effects of hazelnut-derived products on colorectal cancer models.

Author/Year	Sample	Posology/Dosage	Findings
Glei et al., 2018 [9]	Fermented skins of raw and roasted hazelnuts (RC1 = 140.6 °C/25 min, RC2 = 155.1 °C/20 min, RC3 = 180.4 °C/21 min)	2.5% and 5% concentrations	Treatment of HT29 cells with 5% fermented hazelnut skin extracts significantly upregulated SOD2 (3.0-fold increase) and GSTP1 (2.1-fold increase) gene expression, while GPx1 mRNA levels were decreased (0.6-fold reduction). Caspase-3 activity was markedly increased (6.4-fold vs. control, *p* ≤ 0.05). DNA damage induced by H_2_O_2_ exposure was significantly reduced (*p* ≤ 0.05). These results were consistent across both raw and moderately roasted hazelnut skins, suggesting roasting at moderate temperatures does not impair bioactivity. Two-way ANOVA with Bonferroni post hoc analysis confirmed the statistical significance of all main outcomes.
Ramezan et al., 2023 [10]	Hazelnut oil (cold-pressed Iranian cultivar)	Serial dilutions: 1/2, 1/4, 1/8, 1/16	Exposure of HT29 cells to hazelnut oil resulted in significant, dose-dependent reductions in cell viability, with the most pronounced effect observed at 1/2 and 1/4 dilutions (*p* < 0.05). Quantitative PCR analysis revealed downregulation of pro-apoptotic Bax and anti-apoptotic Bcl-2 mRNA levels. Apoptosis was significantly increased based on Annexin V/PI staining (*p* < 0.05). These findings suggest hazelnut oil induces apoptosis through mitochondrial pathways.
Li et al., 2011 [13]	Hazelnut extracts from Oregon and Turkey	6 mg/mL (Oregon), 5 mg/mL (Turkish)	Turkish hazelnut extract inhibited 89% of HT29 cell proliferation after 4 days of treatment, compared to untreated controls. The Oregon extract showed a similar but slightly less potent effect. While MTT assays confirmed inhibition, specific statistical values (e.g., *p*-values) were not reported. Discussion highlighted differences in polyphenolic profiles between cultivars as a potential factor influencing antiproliferative efficacy.
Olofinnade et al., 2021 [16]	Hazelnut extract	IC_50_ values between 459 and 584 μg/mL	Hazelnut extracts induced a significant, dose-dependent inhibition of proliferation in Hela and SK-Mel-28 cancer cells. Apoptosis induction was confirmed by PARP-1 cleavage and Caspase-3 activation after 24–48 h exposure. Although IC_50_ values were provided, detailed *p*-values or statistical analyses were not explicitly reported. The discussion proposed oxidative stress modulation and mitochondrial apoptosis as key mechanisms underlying these effects.
Lux et al., 2012 [17]	Fermented nut samples (hazelnuts)	2.5%, 5%, 10%, and 20% (*v*/*v*)	Treatment of HT29 cells with 20% fermented hazelnut extracts led to a statistically significant reduction in cell proliferation compared to medium control (*p* < 0.05). The reduction was time- and concentration-dependent, with higher doses producing more pronounced effects. The study emphasized the potential role of fermentation in enhancing the anticancer properties of nut-derived bioactives.
Schlörmann et al., 2017 [11]	LT97 colon adenoma cells	Fermented skin (FS) of hazelnuts at 2.5% and 5%	Significant induction of CAT (up to 4.0-fold), SOD2 (up to 2.5-fold), GSTP1 (up to 2.3-fold) mRNA; significant reduction in GPx1 (down to 0.8-fold) (all *p* < 0.05); upregulation of p21 (up to 2.6-fold), downregulation of cyclin D2 (0.4-fold); significant inhibition of LT97 cell growth (down to 46–1.6% depending on dose/time; *p* < 0.05); increased early apoptotic cells (mean +8.4%; *p* < 0.05); increased caspase-3 activity (+4.6-fold; *p* < 0.05).
Güner et al., 2021 [18]	Colon cancer cell line (CaCo-2)	Hazelnut oil at 0.5, 5, and 50 mg/L	Significant antiproliferative activity (IC_50_ = 26.26 ± 3.15 mg/L, *p* < 0.05); significant increase in total antioxidant capacity (TAC) (+49% in CaCo-2 cells at highest dose, *p* < 0.05); significant reduction in total oxidative stress (TOS) (−25% in CaCo-2 cells, *p* < 0.05); strong correlation between MTT viability inhibition and LDH release inhibition (R^2^ = 0.999); inhibition of LDH release by 40% in CaCo-2 cells (*p* < 0.05).

**Table 2:** Risk of bias for in vitro studies investigating the effects of hazelnut-derived products on colorectal cancer models.

Author/Year	Randomization	Blinding	Sample Size Justification	Selective Reporting	Other Bias	Overall Risk of Bias
Glei et al., 2018 [9]	Low	Unclear	Low	Low	Low	Low
Ramezan et al., 2023 [10]	Low	Unclear	Low	Low	Low	Low
Li et al., 2011 [13]	Unclear	Unclear	Unclear	Unclear	Unclear	Unclear
Olofinnade et al., 2021 [16]	Unclear	Unclear	Unclear	Unclear	Unclear	Unclear
Lux et al., 2011 [17]	Unclear	Unclear	Unclear	Unclear	Unclear	Unclear
Schlörmann et al., 2017 [11]	Low	Unclear	Low	Low	Low	Low
Güner et al., 2021 [18]	Low	Unclear	Low	Low	Low	Low

Risk of bias for in vitro studies was assessed using a modified version of the SYRCLE Risk of Bias tool, adapted for preclinical in vitro models. Evaluation domains included randomization procedures, blinding, sample size justification, selective reporting, and other sources of bias. Studies were rated as “Low risk”, “Unclear risk”, or “High risk” based on the reported methodological details.

**Table 3:** Epidemiological studies examining the association between hazelnut consumption and colorectal cancer risk.

Author/Year	Sample	Posology/Dosage	Findings
Nieuwenhuis et al., 2020 [14]	A population-based prospective cohort study in the Netherlands (NLCS) with 120,852 men and women aged 55–69 years.	Measured by a 150-item food frequency questionnaire (FFQ), assessing intake of “other mixed nuts” (“tree nuts”) in the previous year	No association between “tree nut” consumption and CRC molecular subtypes was observed in either sex in categorical analyses. In continuous analyses, the HR (95% CI) per 5 g/d increment of tree nut was 0.52 (0.28–0.95) for MSI tumors in women. This inconsistent finding between categorical and continuous analyses is likely due to chance.

**Table 4:** Risk of bias for the epidemiological study examining the association between hazelnut consumption and colorectal cancer risk.

Author/Year	Selection	Comparability	Outcome Assessment	Follow-Up Adequacy	Overall Risk of Bias
Nieuwenhuis et al., 2020 [14]	Low Risk	Low Risk	Low Risk	Low Risk	Low

Risk of bias for the epidemiological study was evaluated using the Newcastle–Ottawa Scale (NOS), which assesses cohort representativeness, adjustment for confounders, outcome ascertainment, and follow-up adequacy. A global judgment of “Low risk”, “Moderate risk”, or “High risk” was assigned based on the fulfillment of these criteria.

**Table 5:** In vivo studies evaluating the impact of hazelnut-derived products on colorectal carcinogenesis in animal models.

Author/Year	Sample	Posology/Dosage	Findings
Caimari et al., 2015 [12]	*n* = 48 male Golden Syrian hamsters randomly assigned to four groups: standard chow, high-fat diet (HFD), HFD supplemented with FIBEROX^®^ for 8 weeks, or HFD supplemented with FIBEROX^®^ during the final 4 weeks	FIBEROX^®^ (hazelnut skin extract) incorporated into the HFD at 10% w/w for either 8 weeks or 4 weeks	FIBEROX^®^ supplementation reversed the HFD-induced increases in total plasma cholesterol and LDL-cholesterol, significantly reduced circulating free fatty acids and triglycerides, and decreased the lithocholic/deoxycholic bile acid fecal ratio—a recognized biomarker of colon carcinogenesis risk (*p* < 0.05 for all main outcomes). An increased total bile acid excretion was observed, suggesting an enhanced elimination mechanism. Additionally, a significant improvement in the plasma antioxidant capacity and a reduction in plasma malondialdehyde levels were reported in the group supplemented during the final 4 weeks.
Hong et al., 2022 [19]	*n* = 30 21-day-old male Sprague Dawley rats divided into three groups (*n* = 10 per group): control diet without nuts, pistachio-enriched diet (8.1% pistachio), or mixed nuts diet (7.5% mixed nuts, including hazelnuts)	Nut supplementation provided approximately 10% of daily caloric intake over 8 weeks	The mixed nut diet group showed a significant reduction in serum 8-oxo-deoxyguanosine (8-oxo-dG) levels, indicating lower oxidative DNA damage (*p* < 0.05 vs. control). A significant downregulation of the inflammatory gene Rela (NF-κB p65 subunit) in colonic tissue was also observed (*p* < 0.05). Although a decrease in Ptgs2 expression (COX-2) was noted mainly with pistachio intake, no significant differences in colonic cell proliferation (Ki-67 immunostaining) or apoptosis (TUNEL assay) were found between groups. These findings suggest a potential anti-inflammatory and antioxidant protective effect of nut consumption against early events in colorectal carcinogenesis.

**Table 6:** Risk of bias for in vivo studies evaluating the impact of hazelnut-derived products on colorectal carcinogenesis in animal models.

Author/Year	Sequence Generation (Randomization)	Allocation Concealment	Blinding of Investigators	Incomplete Outcome Data	Selective Outcome Reporting	Other Bias	Overall Risk of Bias
Caimari et al., 2015 [12]	Low	Unclear	Unclear	Low	Low	Low	Low
Hong et al., 2022 [19]	Low	Unclear	Unclear	Low	Low	Low	Low

Risk of bias for animal studies was assessed according to the SYRCLE Risk of Bias tool specifically designed for preclinical in vivo experiments. Domains evaluated included sequence generation, allocation concealment, investigator blinding, completeness of outcome data, selective reporting, and other potential biases. Each item was judged as “Low risk”, “Unclear risk”, or “High risk” based on the available methodological information.

## Data Availability

No new data were created or analyzed in this study. Data sharing is not applicable to this article.
